# Editorial: *Cryptosporidium, Giardia, Cyclospora*, and *Toxoplasma* - Insights into their transmission

**DOI:** 10.3389/fcimb.2023.1175108

**Published:** 2023-03-27

**Authors:** Romy Razakandrainibe, Ynes Ortega, Stéphanie La Carbona

**Affiliations:** ^1^ Laboratoire de Parasitologie, EA 7510, Université de Rouen Normandie, Rouen, France; ^2^ Laboratoire de Parasitologie-Mycologie, Centre National de Référence (CNR) Laboratoire Expert Cryptosporidioses, Centre Hospitalier Universitaire de Rouen, Rouen, France; ^3^ Center for Food Safety and Department of Food Science and Technology, University of Georgia, Griffin, GA, United States; ^4^ ACTALIA Food Safety Department, Saint-Lô, France

**Keywords:** *Cryptosporidium*, *Giardia*, *Cyclospora*, *Toxoplasma*, detection, transmission

*Cryptosporidium, Giardia, Cyclospora*, and *Toxoplasma* are four of the most common waterborne and foodborne pathogens that can cause serious health problems in humans. The consequences of these protozoan infections can be devastating, particularly for vulnerable populations such as immunocompromised patients, young children, pregnant women, and the elderly. The zoonotic potential of some protozoan parasites plays a critical role. Preventing their transmission requires a multi-faceted approach, such as (i) educating the public about the dangers of contaminated water and food, through public health campaigns, community outreach programs, and educational materials. (ii) Conducting research and development. Indeed, ongoing research and development can lead to discovering new and effective methods for detecting, treating, and preventing protozoan parasite transmission. (iii) Cross-border collaboration with international organizations can help harmonize standards and regulations and provide access to expertise. (iv) Studying the epidemiology of these protozoans is crucial in understanding the transmission of these parasites. Indeed, a better understanding of transmission patterns and dynamics can help implement effective prevention and control strategies.

Concerning *Giardia duodenalis* transmission, according to Cui et al. China has the world’s largest wild and captive populations of alpine musk deer (*Moschus chrysogaster*). The overall prevalence of *G. duodenalis* in captive alpine musk deer was 19.3% (39/202). Two genetic assemblages, A and E, were identified, for which seven isolates were successfully sequenced at the β-giardin, glutamate dehydrogenase, and triosephosphate isomerase loci. Six isolates harbored assemblage A multi-locus genotypes (MLG), comprising two new MLG subtypes (AI-novel 1 and AI-novel 2). Within assemblage E, no genetic variation was observed. The work emphasizes the potential role of deer in the zoonotic transmission of *G. duodenalis* isolates displayed in the study. In another study, Li et al. showed that of the eight *G. duodenalis*-positive samples (8/826) from nine intensive pig farms in Hubei province (China), seven assemblages E and one assemblage A were identified using β-giardin sequence-based analysis. Among the three sub-assemblages within assemblage A, sub-assemblage AI is most commonly found in animals; humans are more commonly infected with subgroup AII, although subgroup AI has also been seen in some areas or studies (Feng and Xiao, 2011); sub-assemblage AIII is rare and has been found in wild ruminants and some humans giardiasis cases. In the study “*Genomic Comparisons Confirm Giardia duodenalis Sub-assemblage AII as a Unique Species*”, Seabolt et al. present novel signatures of gene content geared toward differential host adaptation and population structuring *via* vertical inheritance. The statistical and biological evidence from the analyzed samples qualifies the sub-assemblages for species rank among the other *G. duodenalis* genetic assemblages. Therefore, these manuscripts raise the importance of zoonotic transmission in the epidemiology of human giardiasis. To answer this question, longitudinal follow-up and subtyping of humans and animals with the same focus on endemicity will establish the sequence of infections in humans and animals.

In the paper “*Divergent Cryptosporidium species and host-adapted Cryptosporidium canis subtypes in farmed minks, raccoon dogs, and foxes in Shandong, China*”, Wang et al. investigated the genetic diversity of *C. canis* and *C. meleagridis* in fur animals. This study collected fecal samples from minks, raccoon dogs, and foxes. Three *Cryptosporidium* species were detected, including *C. canis*, *C. meleagridis*, and *Cryptosporidium* mink genotype. The 60 kDa glycoprotein gene sequencing study in *C. canis* revealed eight subtypes. They were members of two known subtype families, XXa and XXd, and two new subtype families, XXf and XXg, with host adaptation occurring at the subtype family level. *C. canis* from foxes was genetically distinct from *C. canis* from other hosts. Subtype family IIIe (IIIeA21G2R1, IIIeA19G2R1, and IIIeA17G2R1) was found for *C. meleagridis* and two novel subtype families (Xf and Xg) for the *Cryptosporidium mink* genotype. Most cryptosporidiosis in humans is caused by *C. parvum* and *C. hominis*, with the remainder caused by *C. meleagridis*, *C. canis*, and *C. feli*s. The species reported in this study have been identified in infections in immunocompetent and immunosuppressed humans worldwide (McLauchlin et al., 2000; Xiao et al., 2001).

For other animals, in the article entitled “*First Characterization and Zoonotic Potential of Cryptosporidium* spp. *and Giardia duodenalis in Pigs in Hubei Province of China*”, Li et al. investigated the frequency and genotypes of *Cryptosporidium* in pigs in intensive farms in Hubei, China’s central region. The study identified two *Cryptosporidium* species, *C. scrofarum* and *C. suis*. In recent years, these parasites have been found in immunocompetent diarrhea patients and HIV-positive people, indicating that these two pig-adapted *Cryptosporidium* species may be zoonotic.

These two reports indicate that wild and domestic animals might play an important role in transmitting *Cryptosporidium* and still reinforce the public health concern linked to zoonotic potential of *Cryptosporidium* parasites. Establishing and maintaining surveillance systems to track the prevalence and spread of *Cryptosporidium* parasites is critical. These data may be used to detect risk factors and direct public health authorities’ actions.

In their study titled “*Decline in Cryptosporidium Infection in Free-Ranging Rhesus Monkeys in a Park After Public Health Interventions*”, Jia et al. evaluated the effect of intervention measures such as controlling the population of monkeys in efforts to reduce human injuries, feeding monkeys by the public, and reducing damages to the park environment by the animals. Fecal samples from monkeys in Qianling Mountain Park, southwest China, and water samples from park lakes were collected six times between 2013 and 2019. In contrast to the high prevalence of *Cryptosporidium* spp. in fecal samples (10.9%) and water samples (47.8%) in 2010, only 0.7% of fecal samples and 2.4% of water samples were positive for *Cryptosporidium* spp., including *C. hominis* and *C. parvum* in the current study. At subtype level, most prevalent anthroponotic subtypes (IaA13R8, IdA20, IeA11G3T3, and IIcA5G3) were reported before the intervention in 2010 while this study characterized *C. hominis* IfA17G2R3 and *C. parvum* IIdA15G1 and IIpA9. *C. hominis* and *C. parvum* subtypes have transitioned from anthroponotic to zoonotic throughout the long-term study. The latter might have come from other free-roaming creatures in the same park habitat. The shift in prevalent *Cryptosporidium* species/subtypes in rhesus monkeys might be connected to the intervention’s success.

In the last study, titled “*Low prevalence of Toxoplasma gondii in dogs from central China*”, Zhu et al. worked from 2015 to 2021 on fresh dog hearts collected from slaughterhouses; dog blood samples collected from pet hospitals and dog feces; and samples collected from farms, shelters, pet shops, police dog breeding bases, and hospitals. The results showed that *T. gondii* infected 4.29% of dogs. An epidemiological study detected a uniform prevalence of *T. gondii* between humans and dogs, which may be because they lived in the same environment, indicating that dogs may be a suitable sentinel species of *T. gondii* exposure for humans (Tenter et al., 2000). With an estimated 27 million domestic dogs, China makes the third largest in the world and has an unknown number of wild dogs. Environmental pollution due to dog feces is an important exposure way for humans and a public health concern.

In conclusion, preventing protozoan parasite infections requires a comprehensive approach integrating human and animal health, environmental management, and socio-economic development under the One Health umbrella. Raising awareness about the transmission and prevention of protozoan parasites can help reduce the incidence of infections. Proper disposal of human and animal waste, and the provision of clean water, can help prevent the spread of protozoan parasites transmitted through fecal-oral routes. Animal health management can reduce the risk of transmission to humans. Integrated surveillance (One Health approach to surveillance) involves monitoring protozoan parasite infections in both humans and animals, as well as in the environment, to detect outbreaks early and implement appropriate control measures. Implementing these strategies can help reduce the burden of protozoan parasite infections on human and animal populations.

## Author contributions

All authors listed have made a substantial, direct, and intellectual contribution to the work and approved it for publication.
